# Early symptoms and 12-week follow-up of pediatric omicron infections during the Beijing outbreak

**DOI:** 10.3389/fped.2025.1389572

**Published:** 2025-08-05

**Authors:** Qin Hui, Jing Liu, Hua Fan, Xiaonan Hou, Xuan Li, Wei Li, Qi Zhang

**Affiliations:** Department of Pediatrics, China-Japan Friendship Hospital, Beijing, China

**Keywords:** children, omicron, symptom, COVID-19, Beijing

## Abstract

**Objectives:**

To investigate the epidemiological and clinical characteristics of children infected with the Omicron variant of SARS-CoV-2 during the early outbreak in Beijing, with particular attention to symptom profiles, clinical management, and persistent symptoms at 12 weeks post-infection.

**Methods:**

This prospective study enrolled children under 18 years of age with confirmed or suspected Omicron infection in Beijing between December 2022 and January 2023. Data were collected via an online questionnaire targeting both community-managed and outpatient children. A follow-up survey was conducted at 12 weeks post-infection to assess long-term symptoms.

**Results:**

A total of 1,610 children aged 15 days to 18 years were included (median age: 3.00 years; 51.4% boys). Fever (96.4%) was the most common symptom, with a mean peak temperature of 39°C (range: 37.6–41°C). Other frequent symptoms included cough (59.1%), runny nose (43.7%), and fatigue (22.1%). The mean duration of fever was 2.05 ± 1.09 days, and the mean duration of all symptoms was 5.89 ± 4.35 days, with both showing significant differences across age groups (*p* < 0.001). By one week post-infection, symptoms had resolved in 34.2% of cases. At 12 weeks, 2.9% (43/1,471) of children still reported persistent symptoms. The most common were cough (1.4%), nasal congestion (1.1%), dry throat and exertional dyspnea (each 0.7%), and fatigue (0.6%).

**Conclusions:**

Omicron infection affected children across all age groups, with a higher prevalence in younger children. Fever and cough were the predominant acute symptoms, while a small subset reported mild persistent symptoms 12 weeks post-infection.

## Introduction

The novel coronavirus disease 2019 (COVID-19), caused by severe acute respiratory syndrome coronavirus 2 (SARS-CoV-2), remains a significant global public health challenge. As of December 23, 2022, an estimated over 650 million COVID-19 cases were confirmed worldwide, causing 6.65 million deaths (1–3). On November 24, 2021, the Omicron (B.1.1.529) mutant strain was first identified in South Africa, and spread quickly around the world (4, 5). In January 2022, the Omicron variant has almost replaced the Delta variant, triggering the fifth wave of the global COVID-19 outbreak (6, 7). In the United States, the Omicron infection is highly prevalent, especially among children (8). In December 2022, Omicron BF.7 and its subvariants spread rapidly in Beijing, leading to a spike in pediatric COVID-19 cases. Recent studies have examined the clinical characteristics of pediatric Omicron infections, reporting generally milder disease severity compared to earlier variants (9, 10). However, these studies focused primarily on acute clinical manifestations and were limited to specific regions, providing limited data on long-term outcomes and regional variations.

Persistent symptoms, or “long COVID,” are increasingly recognized in both adults and children, though the evidence base for pediatric populations remains limited (11). According to the National Institute for Health and Care Excellence (NICE), long COVID encompasses symptoms lasting or newly appearing beyond 12 weeks post-infection. In contrast, the early features of Omicron infection—defined as acute symptoms occurring within the first two weeks post-infection—may differ across age groups and vaccination status, and understanding these patterns is essential for tailoring public health interventions. The currently prevailing Omicron mutant strains are highly infectious, and are characterized by immune escape and superinfection capabilities (7, 12, 13). Children are high risk populations for Omicron virus, with varying clinical symptoms, especially after experiencing 3-year immunity gap. Accurate population data are scant on the early epidemiological and clinical features of pediatric Omicron infection in December 2022.

To address these gaps, we conducted a large-scale study of children infected with the Omicron variant during the early outbreak in Beijing (December 2022–January 2023). Our study aimed to characterize both the early-phase clinical manifestations and the persistent symptoms consistent with long COVID observed at 12 weeks post-infection. By comparing our findings with previous studies and highlighting regional differences, we aim to provide novel insights into the clinical course of Omicron infection in children.

## Methods

### Study participants

This was a prospective, observational study targeting pediatric patients (<18 years old) residing in all 16 districts of Beijing. Children were enrolled if they were infected with the Omicron variant of SARS-CoV-2 between December 1, 2022, and January 31, 2023. SARS-CoV-2 infection was confirmed either by positive antigen or polymerase chain reaction (PCR) test from a respiratory specimen, or through self-report, defined as having typical COVID-19 symptoms with a known exposure history during the peak of the local outbreak. This definition aligns with regional epidemiological surveillance standards during the Omicron BF.7 wave. Symptom profiles were stratified by age groups.

The conduct of this study was reviewed and approved by the Ethics Committee of China-Japan Friendship Hospital, and the committee waived the need for informed consent from study participants in this questionnaire-based COVID-19 symptom survey.

### Data collection

Data were collected using an online questionnaire platform and administered in two stages: Initial survey (December 2022–January 2023): participants included two subgroups: (1) parents of children infected with Omicron who were managed at home or in the community, and (2) parents of pediatric patients who sought outpatient care at the Department of Pediatrics, China-Japan Friendship Hospital. The electronic questionnaire was distributed via official hospital systems and parent-teacher communication channels (e.g., WeChat groups), enabling broad coverage of both clinical and community populations. Follow-up survey (March–April 2023): Conducted approximately 12 weeks after infection, this survey aimed to identify persistent or newly emerged symptoms. The follow-up cohort was not strictly limited to those who completed the initial survey, although there was partial overlap. The survey was open to all families of children previously infected with Omicron during the defined outbreak window. The content of both the initial and follow-up questionnaires is provided as Supplementary Material. The survey captured a wide range of variables, including: demographic characteristics (age, sex), SARS-CoV-2 diagnostic method (antigen test, RT-PCR, or self-report), COVID-19 vaccination status, pre-existing comorbidities, acute symptoms, persistent symptoms (at 12 weeks post-infection), treatment or healthcare utilization (home care, outpatient visit, emergency visit, hospitalization, ICU admission).

### Statistical analyses

The statistical analysis was performed using the software SPSS (IBM Statistics 23.0). Categorical variables are described as count with percentage. Continuous variables in normal distribution are described as mean (standard deviation), and as median (interquartile range) otherwise. Group comparisons of categorical variables were performed using the Chi-squared (*χ*^2^) test or Fisher's exact test, as appropriate. For continuous variables, differences between groups were assessed using the Student's *t*-test or one-way analysis of variance (ANOVA) for normally distributed data, and the Mann–Whitney *U* test or Kruskal–Wallis test for non-parametric data. Two-sided *P* value less than 0.05 is considered to be statistically significant (1, 2, 14).

## Results

### Baseline characteristics

Total 1,610 children infected by Omicron virus aged 15 days to 18 years were eligible for inclusion in this study. The median age of study children was 3.00 (interquartile range: 0.92, 6.50) years, and 51.4% of them were boys. The baseline characteristics of study children are presented in Table 1. Of all Omicron-related comorbidities, allergic rhinitis was the most prevalent, accounting for 14.7%, followed by febrile convulsion (2.5%) and asthma (1.3%). The prevalence of comorbidities varied across age groups. Allergic rhinitis and asthma were significantly more common in older children, particularly in the 6–11 years and 12–18 years groups, while febrile convulsion was predominantly observed in younger children, especially in the 3–5 years group. 42.5% of children had Omicron infection confirmed by COVID-19 antigens, and this proportion increased with aging.

**Table 1:** Basic characteristics of study children infected by omicron infection.

Characteristics	Total	<1 year	1–2 years	3–5 years	6–11 years	12–18 years	*p*-value
Number	1,610	419	322	344	415	110	
Age (years)	3.00 [0.92, 6.50]	6.00 [4.00, 8.01]^a^	1.50 [1.00, 2.00]	4.00 [3.50, 5.00]	8.00 [6.00, 9.00]	14.00 [12.00, 15.00]	<0.001
Male (%)	827 (51.4%)	215 (51.3)	168 (52.2)	181 (52.6)	208 (50.1)	55 (50.0)	0.959
Comorbidities							
Allergic rhinitis (%)	237 (14.7)	2 (0.5)	10 (3.1)	70 (20.3)	123 (29.6)	32 (29)	<0.001
Asthma (%)	21 (1.3)	0 (0.0)	0 (0.0)	6 (1.7)	10 (2.4)	5 (4.5)	<0.001
Cardiovascular disease (%)	4 (0.2)	1 (0.2)	1 (0.3)	0 (0.0)	2 (0.5)	0 (0.0)	0.8
Epilepsy (%)	3 (0.2)	0 (0.0)	1 (0.3)	0 (0.0)	0 (0.0)	2 (1.8)	0.003
Metabolic disorder (%)	1 (0)	0 (0.0)	0 (0.0)	1 (0.3)	0 (0.0)	0 (0.0)	0.482
Gastrointestinal (%)	4 (0.2)	1 (0.2)	1 (0.3)	0 (0.0)	2 (0.5)	0 (0.0)	0.8
Congenital (%)	4 (0.2)	0 (0.0)	0 (0.0)	2 (0.6)	2 (0.5)	0 (0.0)	0.401
Cancer (%)	2 (0.1)	0 (0.0)	0 (0.0)	0 (0.0)	2 (0.5)	0 (0.0)	0.284
Rheumatic (%)	1 (0)	0 (0.0)	0 (0.0)	0 (0.0)	1 (0.2)	0 (0.0)	0.74
Febrile convulsion (%)	40 (2.5)	1 (0.2)	4 (1.2)	12 (3.5)	18 (4.3)	5 (4.5)	<0.001
COVID-19 determination							
Covid-19 Antigen-positive (%)	684 (42.5)	99 (23.6)	102 (31.7)	163 (47.4)	246 (59.3)	74 (67.3)	<0.001
Covid-19 Nucleic acid-positive (%)	52 (3.2)	21 (5.0)	4 (1.2)	5 (1.5)	19 (4.6)	3 (2.7)	0.005
Self-report (%)	874 (54.3)	156 (37.2)	127 (39.4)	135 (39.2)	130 (31.3)	26 (23.6)	0.005

^a^ The unit of measurement is month. Data are expressed as median (interquartile range) or count (percentage), where appropriate.

### Symptom manifestations

The main symptoms of children with Omicron infection are summarized in Table 2. Of the 1,610 cases, 1,552 (96.4%) suffered fever (axillary temperature >37.5℃), with a mean fever spike at 39℃ (range: 37.6–41℃). Other main symptoms in children infected with Omicron were cough (*n* = 952, 59.1%), runny nose (*n* = 704, 43.7%), fatigue (*n* = 357, 22.1%), sore throat (*n* = 335, 20.8%), headache (*n* = 234, 14.5%) (Table 2).

**Table 2:** Main symptoms of study children infected by omicron infection.

Symptoms	Total	<1 year	1–2 years	3–5 years	6–11 years	12–18 years	*p*-value
Fever (%)	1,552 (96.4)	404 (96.4)	317 (98.4)	329 (95.6)	398 (95.9)	104 (94.5)	0.211
Temperature peak (℃)	39.20 [38.80, 39.70]	39.00 [38.60, 39.50]	39.50 [39.00, 40.00]	39.20 [38.80, 39.80]	39.20 [38.80, 39.70]	39.00 [38.50, 39.50]	<0.001
Sore throat (%)	335 (20.8)	46 (11.0)	42 (13.0)	58 (16.9)	136 (32.8)	53 (48.2)	<0.001
Hoarseness (%)	229 (14.2)	101 (24.1)	53 (16.5)	23 (6.7)	40 (9.6)	12 (10.9)	<0.001
Runny nose (%)	704 (43.7)	197 (47.0)	131 (40.7)	146 (42.4)	176 (42.4)	54 (49.1)	0.301
Cough (%)	952 (59.1)	261 (62.3)	165 (51.2)	191 (55.5)	253 (61.0)	82 (74.5)	<0.001
Dyspnea (%)	22 (1.4)	10 (2.4)	2 (0.6)	4 (1.2)	5 (1.2)	1 (0.9)	0.371
Wheeze (%)	69 (4.2)	32 (7.6)	16 (5.0)	6 (1.7)	10 (2.4)	5 (4.5)	<0.001
Headache (%)	234 (14.5)	8 (1.9)	20 (6.2)	28 (8.1)	130 (31.3)	48 (43.6)	<0.001
Convulsions (%)	31 (1.9)	10 (2.4)	11 (3.4)	6 (1.7)	4 (1.0)	0 (0.0)	0.082
Heterosmia (%)	34 (2.1)	1 (0.2)	1 (0.3)	5 (1.5)	17 (4.1)	10 (9.1)	<0.001
Hypogeusia (%)	60 (3.7)	6 (1.4)	5 (1.6)	9 (2.6)	21 (5.1)	19 (17.3)	<0.001
Fatigue (%)	357 (22.1)	50 (11.9)	66 (20.5)	77 (22.4)	116 (28.0)	48 (43.6)	<0.001
Muscular soreness (%)	180 (11.2)	3 (0.7)	19 (5.9)	32 (9.3)	81 (19.5)	45 (40.9)	<0.001
Rash (%)	82 (5.1)	42 (10.0)	15 (4.7)	10 (2.9)	13 (3.1)	2 (1.8)	<0.001
Vomit (%)	204 (12.7)	51 (12.2)	52 (16.1)	32 (9.3)	63 (15.2)	6 (5.5)	0.005
Abdominal pain (%)	78 (4.8)	9 (2.1)	11 (3.4)	26 (7.6)	26 (6.3)	6 (5.5)	0.004
Diarrhea (%)	188 (11.7)	86 (20.5)	38 (11.8)	21 (6.1)	35 (8.4)	8 (7.3)	<0.001
Chest pain (%)	8 (0.5)	0 (0.0)	0 (0.0)	1 (0.3)	4 (1.0)	3 (2.7)	0.003
Chest tightness (%)	16 (1)	0 (0.0)	1 (0.3)	2 (0.6)	8 (1.9)	5 (4.5)	<0.001

Data are expressed as median (interquartile range) or count (percentage), where appropriate.

Temperature peak was higher in children 1–2 years old than other age groups (*p* < 0.01). Higher incidence of hoarseness (*n* = 101, 24.1%), wheeze (*n* = 32, 7.6%), rash (*n* = 42, 10%), diarrhea (*n* = 86, 20.5%) were observed in infants (*p* < 0.01). The incidence of abdominal pain (*n* = 78, 4.8%) was highest in pre-school aged children (3–5 years) (*p* < 0.01). Cough (*n* = 82, 74.5%), sore throat (*n* = 53, 48.2%), headache (*n* = 48, 43.6%), heterosmia (*n* = 10, 9.1%), hypogeusia (*n* = 19, 17.3%), fatigue (*n* = 48, 43.6%), muscle soreness (*n* = 45, 40.9%), chest pain (*n* = 3, 2.7%), and chest tightness (*n* = 5, 4.5%) were more frequently seen in adolescents (12–18 years) relative to other age groups (*p* < 0.01) (Table 2).

### Duration of symptoms and outcomes

The duration of fever and symptoms differed significantly across age groups (*p* < 0.001). The mean duration of fever was 2.05 ± 1.09 days, and the mean duration of overall symptoms was 5.89 ± 4.35 days (Table 3). When stratified by age, the average duration of symptoms was longest in adolescents aged 12–18 years (7.11 ± 4.26 days) and children under 1 year (6.77 ± 4.95 days), compared to shorter durations in children aged 1–3 years (5.65 ± 3.71 days), 3–6 years (5.15 ± 4.15 days), and 6–12 years (5.53 ± 4.23 days). Similarly, although the median fever duration was consistently 2 days across all groups, the mean fever duration showed statistically significant variation, ranging from 1.94 ± 1.11 days in infants (<1 year) to 2.27 ± 0.95 days in toddlers (1–3 years).

**Table 3:** Duration and outcomes of study children infected by omicron infection.

Duration and outcomes	All	<1 year	1–3 years	3–6 years	6–12 years	12–18 years	*p*-value
Number	1,610	419	322	344	415	110	
Duration of fever (days)	2.05 ± 1.09	1.94 ± 1.11	2.27 ± 0.95	2.00 ± 1.04	2.02 ± 1.24	2.15 ± 0.93	<0.001
Duration of symptoms (days)	5.89 ± 4.35	6.77 ± 4.95	5.65 ± 3.71	5.15 ± 4.15	5.53 ± 4.23	7.11 ± 4.26	<0.001
Therapy options							
Home therapy	1,470 (91)	351 (83.8)	289 (89.8)	326 (94.8)	397 (95.7)	107 (97.3)	<0.001
Outpatient clinic visit	67 (4.1)	28 (6.7)	17 (5.3)	9 (2.6)	12 (2.9)	1 (0.9)	0.018
Emergency department visit	47 (2.9)	24 (5.7)	11 (3.4)	6 (1.7)	4 (1.0)	2 (1.8)	<0.001
Hospitalization	23 (1.4)	14 (3.3)	5 (1.6)	2 (0.6)	2 (0.5)	0 (0.0)	0.003
ICU admission	3 (0.2)	2 (0.5)	0 (0.0)	1 (0.3)	0 (0.0)	0 (0.0)	0.468

Data are expressed as median (interquartile range) or count (percentage), where appropriate.

Total 1,470 (91%) children were treated at home, and this trend was more obvious in older age groups. Among children under 3 years old, the proportion of receiving treatment in outpatient clinics was relatively high, reaching 6.7% for children aged <1 year and 5.3% for children aged 1–3 years. Meanwhile, 5.7% and 3.4% of them chose emergency department visits (Table 3).

A total of 23 children (1.4%) were hospitalized, with three requiring admission to the Intensive Care Unit (ICU) due to dyspnea. Hospitalization was most common in infants under 1 year (3.3%) and children aged 1–3 years (1.6%), while no hospitalizations occurred in the 12–18 year group (Table 3).

About 34.2% of children's symptoms disappeared within one week. The top three common symptoms that still presented after one week were cough, fatigue, and loss of appetite, accounting for 44%, 8.2%, and 16.4% of study children, respectively, and they differed significantly across age groups (*p* < 0.01, Table 4).

**Table 4:** Symptoms after a week in study children infected by omicron infection.

Symptoms	All	<1 year	1–3 years	3–6 years	6–12 years	12–18 years	*p*-value
Number	1,610	419	322	344	415	110	
Cough (%)	709 (44)	208 (49.6)	105 (32.6)	128 (37.2)	200 (48.2)	68 (61.8)	<0.001
Hoarseness (%)	119 (7.3)	59 (14.1)	27 (8.4)	16 (4.7)	13 (3.1)	4 (3.6)	<0.001
Chest tightness (%)	13 (8)	0 (0.0)	2 (0.6)	1 (0.3)	5 (1.2)	5 (4.5)	<0.001
Chest pain (%)	6 (0.4)	0 (0.0)	0 (0.0)	0 (0.0)	5 (1.2)	1 (0.9)	0.007
Headache (%)	25 (1.6)	2 (0.5)	1 (0.3)	3 (0.9)	10 (2.4)	9 (8.2)	<0.001
Fatigue (%)	131 (8.2)	22 (5.3)	26 (8.1)	16 (4.7)	37 (8.9)	30 (27.3)	<0.001
Appetite loss (%)	264 (16.4)	99 (23.6)	79 (24.5)	40 (11.6)	35 (8.4)	11 (10.0)	<0.001
Diarrhea (%)	75 (4.7)	41 (9.8)	10 (3.1)	2 (0.6)	14 (3.4)	6 (5.5)	<0.001
Abdominal pain (%)	17 (1.1)	5 (1.2)	2 (0.6)	4 (1.2)	6 (1.4)	0 (0.0)	0.766
Asymptomatic (%)	550 (34.2)	111 (26.5)	119 (37.0)	145 (42.2)	148 (35.7)	27 (24.5)	<0.001

Data are expressed as median (interquartile range) or count (percentage), where appropriate.

### Vaccination status

As shown in Table 5, 692 of children had received at least one dose of an inactivated COVID-19 vaccine, accounting for 79.6% of total 869 pediatric cases aged ≥3 years who are eligible for COVID-19 vaccines. Of 869 vaccine-eligible children, 177 (20.4%) were unvaccinated, 45 (5.2%) had received one dose, and 647 (74.4%) had received two doses. Among 344 pre-school aged children 3–5 years, the proportions of one dose and two doses of COVID-19 vaccination were 9% (31/344) and 53.8% (185/344), respectively. The vaccination rate of school-age children (6–12 years) followed an increasing trend, with the proportions of one dose and two doses of COVID-19 vaccination being 2.9% (12/415) and 85.5% (355/415). Noteworthily, 90% (99/110) of teenagers were vaccinated two doses of inactivated COVID-19 vaccines.

**Table 5:** Vaccine status of study children infected by omicron infection.

Vaccination	Total	3–5 years	6–11 years	12–18 years	*p*-value
Vaccinated	692 (79.6)	216 (62.8)	367 (88.4)	100 (90.9)	NA
1 Dose	45 (5.2)	31 (9.0)	12 (2.9)	1 (0.9)	NA
2 Doses	647 (74.4)	185 (53.8)	355 (85.5)	99 (90)	NA

Data are expressed as median (interquartile range) or count (percentage), where appropriate.

Therapeutic options varied notably by vaccination status. Home-based care was significantly more common among vaccinated children (96.2%) than unvaccinated children (87.5%). Conversely, unvaccinated children were more likely to require medical intervention, including outpatient clinic visits (5.7% vs. 2.2%), emergency department visits (4.1% vs. 1.3%), hospitalization (2.3% vs. 0.3%), and ICU admission (0.3% vs. 0.0%) (all *p*-values < 0.001). These results are summarized in Supplementary Table S1.

### Follow-up survey

**Figure 1:**
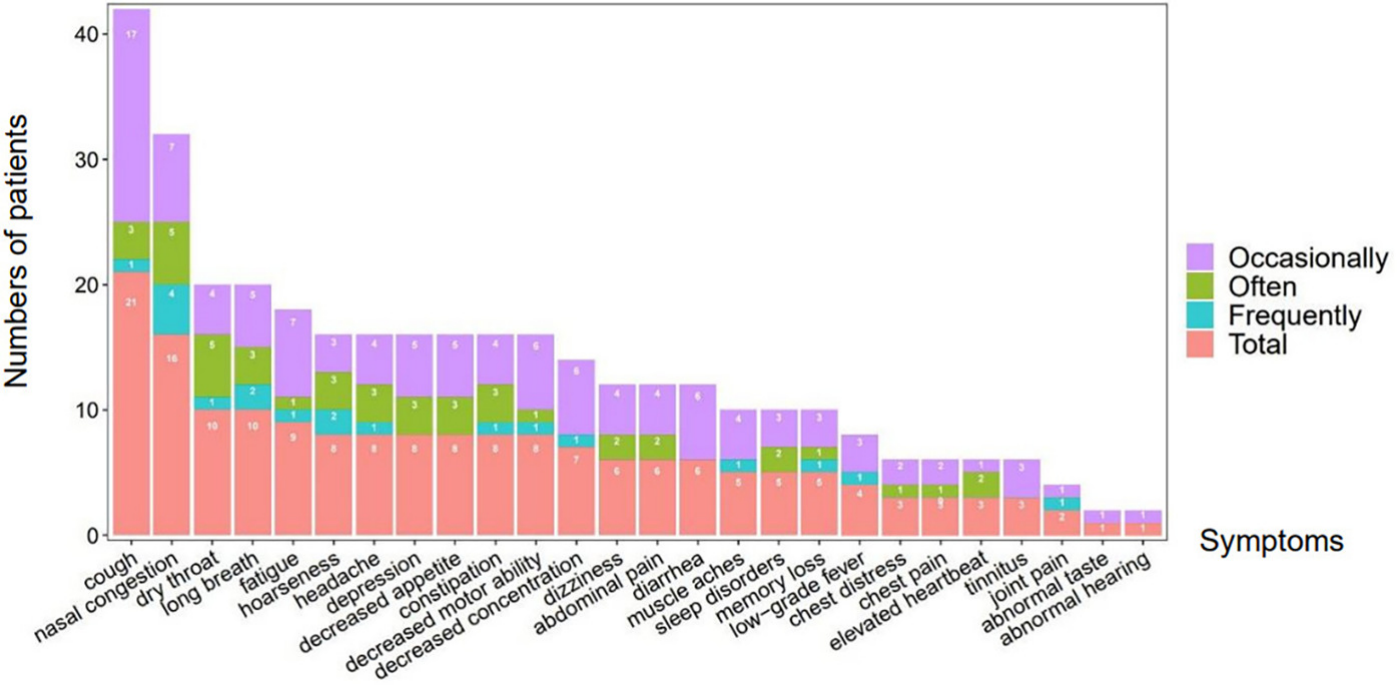
Distribution of persistent symptoms in children 12 weeks after omicron infection.

A total of 1,471 children infected with the Omicron variant were followed up at 12 weeks post-infection. The median age was 7 years (interquartile range: 5–9), and 826 (56.2%) were boys. Among them, 43 children (2.9%) reported at least one persistent or newly emerged symptom, consistent with the definition of long COVID. Figure 1 provide detailed distributions of these symptoms across severity levels (“occasionally,” “often,” and “frequently”).

The most frequently reported symptom was cough (1.4%), followed by nasal congestion (1.1%), dry throat and exertional dyspnea (0.7% each), and fatigue (0.6%). Other symptoms included headache, decreased appetite, gastrointestinal disturbances (e.g., constipation, abdominal pain, diarrhea), and neuropsychological complaints such as decreased concentration, depression, dizziness, and memory loss.

## Discussion

To the best of our knowledge, this is the first study that has described the epidemiological and clinical features of early-stage Omicron variant infection among children in the Beijing outbreak from December 2022 to January 2023. Our findings indicate that children of all age groups, from 15 days to 18 years, were susceptible to Omicron infection, with the highest proportion observed among younger children. Fever and cough were the most common symptoms during the acute phase, and most children experienced mild illness.

Consistent with global reports, Omicron infection has impacted children across all age groups (15, 16). In our cohort, the median age of infected children was 3 years—lower than reported in similar studies from Shanghai (5 years) (17) (median age: 5 years) and Changchun (18) (median age: 5.7 years),suggesting a higher vulnerability in younger children. Previous data from the Journal of the American Medical Association and The Lancet preprint server suggested that Omicron cases skew younger, with increased hospitalization rates in individuals under 20 during the Omicron wave compared to Delta (19–22). These findings underscore the importance of vigilance in managing pediatric populations during Omicron-dominant periods.

Despite the high infection rate among younger age groups, symptom severity remained mild for most children. This may reflect both the lower pathogenicity of the Omicron variant and widespread vaccination coverage among children aged ≥3 years in China (5, 23, 24). In our study, we further explored the relationship between vaccination status and disease severity. Among 1,610 children with Omicron infection, 43.0% had received at least one dose of a COVID-19 vaccine. Notably, unvaccinated children were more likely to require medical services, including outpatient visits, emergency department visits, hospitalization, and ICU admission. These findings suggest that COVID-19 vaccination offers protective benefits against severe clinical outcomes in pediatric Omicron infection, particularly among children over 3 years old. Recent immunological studies support this evidence. For instance, Cinicola et al. demonstrated that the BNT162b2 mRNA vaccine effectively induced both humoral and cellular immune memory in children aged 5–11 years, including strong responses against the Omicron variant. Importantly, children with prior undetected infection developed stronger and broader immune responses after vaccination, reflecting the benefit of hybrid immunity (25). Similarly, Capponi et al. found the vaccine to be well tolerated, immunogenic, and effective in preventing severe COVID-19 outcomes in children, reinforcing its public health value (26). Together, these data reinforce the importance of COVID-19 vaccination in children as a public health strategy.

Regarding symptom presentation, 96% of symptomatic cases in our cohort presented with fever. This is notably higher than during the first COVID-19 wave in Wuhan (58%) and the United States (56%) (27). The difference may be attributed to our study's focus on symptomatic individuals. The average duration of fever was short (1.7 days), and fever (51%) and cough (41%) were the most frequently reported symptoms—rates lower than those observed in adults.

As the incidence of SARS-CoV-2 infection increases, a growing concern arises on persistent multiorgan symptoms after acute infection, commonly termed as ‘long COVID’. While extensive data are available for adults, pediatric long COVID remains underexplored. It is reported that 4%–66% of children experienced post-acute COVID-19 symptoms, including insomnia, respiratory symptoms, nasal congestion, fatigue, muscle and joint pain, concentration difficulties, and loss of smell and taste (15, 28, 29). In our study, 2.9% of children reported persistent symptoms 12 weeks after Omicron infection, a rate that falls within the lower bound of the wide range reported in prior studies. Behnood et al. conducted a systematic review and meta-analysis of 22 studies and found that the most commonly reported symptoms included fatigue, diarrhoea, and loss of smell (30). In comparison, the most frequent symptoms in our cohort were cough (1.4%), nasal congestion (1.1%), dry throat (0.7%), fatigue (0.6%), and neurocognitive symptoms such as decreased concentration and depression (each <1%). These findings suggest that while the prevalence of long COVID in children infected with Omicron may be lower than in earlier variants, persistent symptoms still occur and may impact multiple systems.

### Limitations

Several limitations should be acknowledged in this study. First, the data were collected through online questionnaires, which introduces the possibility of recall bias and reporting inaccuracies. Second, the initial and follow-up surveys targeted two partially overlapping but non-identical populations, meaning that the cohort used to assess persistent symptoms at 12 weeks was not strictly the same as the one used for acute-phase analysis. This may limit the ability to track individual symptom trajectories over time. Finally, all participants were children residing in Beijing, China, so the findings may not be generalizable to pediatric populations of different ethnic or regional backgrounds.

## Conclusions

In this large prospective study of children infected with the Omicron variant in Beijing, we found that all pediatric age groups were susceptible to infection, with the highest proportion observed among younger children. Fever and cough were the most common acute symptoms, and the overall clinical course was mild in most cases. Symptom duration and healthcare utilization varied significantly by age group. Although the majority of children recovered within one week, 2.9% reported persistent symptoms at 12 weeks post-infection, most commonly cough, nasal congestion, and fatigue. These findings underscore the need for continued monitoring of long COVID in pediatric populations.

## Data Availability

The raw data supporting the conclusions of this article will be made available by the authors, without undue reservation.

## Supplementary material

Supplementary Table S1 Comparison of clinical management by vaccination status.
