# Supplementary material for: Early symptoms and 12-week follow-up of pediatric omicron infections during the Beijing outbreak
Source: Front Pediatr. 2025 Aug 5;13:1389572. doi: 10.3389/fped.2025.1389572 (PMC12361124; doi:10.3389/fped.2025.1389572)
Supplement: Supplementary Table S1 — Comparison of clinical management by vaccination status. [file Datasheet2.pdf]

Questionnaire of COVID-19 12-week Follow-up for Children

Post-COVID-19 Condition (PCC), commonly referred to as long COVID, refers to the disease that occurs in individuals with a possible or confirmed history of COVID-19 infection. This condition typically develops within three months after the onset of COVID-19 symptoms and lasts for at least two months. These symptoms and effects cannot be explained by other diagnoses. According to the World Health Organization (WHO), while most individuals infected with COVID-19 recover fully, some may experience a variety of long-term symptoms, such as fatigue, shortness of breath, and cognitive dysfunction.

Currently, the occurrence of long COVID symptoms in children is not well understood. We invite you to participate in our survey on the long-term symptoms in children after COVID-19 infection. This is an anonymous survey, and the data will be used for scientific research only. Please feel free to fill it out, as it will contribute to joint efforts in promoting children's health. Thank you for your support.

---

1. Date of Birth of the Child:

[Open-ended question]

---

2. Gender of the Child:

[Single-choice question]
○ Male
○ Female

3. Height and Weight of the Child:

[Matrix question]

- Height (cm): ________________________
- Weight (kg): ________________________

4. Date When the Child First Showed Symptoms of COVID-19:

[Single-choice question]
○ Early December
○ Mid-December
○ Late December
○ After January

5. Did the Child Have a Fever During COVID-19 Infection?

[Single-choice question]

○ No (Please skip to question 8)

○ Yes

6. Duration of Fever After COVID-19 Infection (Half-day is filled as 0.5, and so on):

[Open-ended question]

---

7. What Was the Highest Recorded Temperature During COVID-19 Infection?

[Open-ended question]

---

8. Was COVID-19 Related Testing Conducted for the Child?

[Multiple-choice question]

□ Positive antigen test for the child

□ Positive nucleic acid test for the child

□ The child was not tested

□ Positive antigen or nucleic acid test in cohabitants within one week before or after onset

□ No testing was done for the whole family

9. How Many Days Did It Take for the Child's Antigen or Nucleic Acid Test to Turn Negative?

[Single-choice question]

○ Not monitored

○ Monitored for ____ days

10. Has the Child Been Vaccinated Against COVID-19?

[Single-choice question]

○ Not vaccinated under 3 years old

○ Not vaccinated over 3 years old due to safety concerns

○ Not vaccinated over 3 years old due to underlying health conditions

○ Not vaccinated over 3 years old due to repeated illness

○ Completed 1 dose

○ Completed 2 doses

○ Completed 3 doses (booster)

11. Does the Child Have Any of the Following Underlying Health Conditions?

[Multiple-choice question]

□ Premature birth
□ Twin or multiple births
□ Anemia
□ Picky eater, poor appetite, or feeding difficulties
□ Allergic rhinitis
□ Asthma
□ Eczema
□ Febrile convulsions
□ Epilepsy
□ Heart disease
□ Metabolic disorders
□ Chronic gastrointestinal diseases
□ Congenital diseases
□ Cancer
□ Rheumatic diseases
□ Endocrine disorders
□ Blood system diseases
□ Developmental delays
□ Autism
□ None

12. Does the Child Currently Have Any Symptoms After COVID-19 Infection?

[Single-choice question]
○ No (Please skip to the end of the questionnaire and submit)
○ Yes

13. Which Symptoms Have Not Resolved Since the First Infection (Not Including Symptoms That Appeared During This Visit)?

[Matrix single-choice question]

**Symptom**	**None**	**Occasionally**	**Frequently**	**Constantly**
Cough	○	○	○	○
Low-grade fever (37°C < temperature < 37.5°C)	○	○	○	○
Dry throat	○	○	○	○
Hoarseness	○	○	○	○

**Symptom**	**None**	**Occasionally**	**Frequently**	**Constantly**
Nasal congestion	○	○	○	○
Headache	○	○	○	○
Dizziness	○	○	○	○
Fatigue	○	○	○	○
Muscle soreness	○	○	○	○
Joint pain	○	○	○	○
Sleep disturbances	○	○	○	○
Decreased attention	○	○	○	○
Decreased memory	○	○	○	○
Low mood / Crying	○	○	○	○
Decreased appetite	○	○	○	○
Abdominal pain	○	○	○	○
Diarrhea	○	○	○	○
Constipation	○	○	○	○
Chest tightness	○	○	○	○
Chest pain	○	○	○	○
Shortness of breath	○	○	○	○
Palpitations	○	○	○	○
Decreased exercise ability	○	○	○	○
Olfactory dysfunction	○	○	○	○
Gustatory dysfunction	○	○	○	○
Auditory dysfunction	○	○	○	○

Symptom	None	Occasionally	Frequently	Constantly
Tinnitus	○	○	○	○
