# Supplementary material for: Early symptoms and 12-week follow-up of pediatric omicron infections during the Beijing outbreak
Source: Front Pediatr. 2025 Aug 5;13:1389572. doi: 10.3389/fped.2025.1389572 (PMC12361124; doi:10.3389/fped.2025.1389572)
Supplement: Supplementary file 2 [file Datasheet1.pdf]

Questionnaire of COVID-19 Infection Survey for Children

Recent variants of the COVID-19 virus have spread widely across the country, and children have become a susceptible population. Although most children experience mild symptoms, the absolute number of severe cases in children is still concerning, given the large population in China. The continuous mutation of the virus has also introduced new challenges. To better understand the characteristics of children infected with COVID-19 and to follow up on potential long-term impacts of infection, we invite you to participate in this survey. Your participation will help promote children's health efforts during the pandemic. (This survey is anonymous, and the privacy of patients will be protected. Please feel free to complete it.)

---

1. Gender of the Child:

[Single-choice]
○ Male
○ Female

2. Age of the Child:

[Open-ended]
Please specify the age in months for children under 1 year and in days for children under 1 month (e.g., 1 year, 1 month, 1 day).

---

3. Symptoms Experienced by the Child:

[Multiple-choice]
☐ Sore throat
☐ Hoarseness
☐ Nasal congestion, runny nose
☐ Cough
☐ Shortness of breath
☐ Wheezing
☐ Headache
☐ Seizures
☐ Olfactory dysfunction
☐ Gustatory dysfunction
☐ Fatigue
☐ Muscle soreness

☐ Rash
☐ Vomiting
☐ Abdominal pain
☐ Diarrhea
☐ Chest pain
☐ Chest tightness
☐ Other: ______________
☐ Fever

4. Duration of Intermittent Fever (Days)

[Open-ended]

---

5. Highest Recorded Temperature (°C)

[Open-ended]

---

6. Did the Child Seek Medical Care?

[Single-choice]
○ Home treatment
○ Outpatient care
○ Emergency care
○ Hospitalization
○ Intensive Care Unit (ICU) treatment

7. Has the Child Been Vaccinated for COVID-19?

[Single-choice]
○ No
○ Completed 1 dose
○ Completed 2 doses
○ Completed 3 doses
○ Last vaccination date: ______________

8. Medications Taken During Illness:

[Multiple-choice]
☐ Ibuprofen
☐ Acetaminophen
☐ Traditional Chinese medicine

☐ Compound cold medicine

☐ Nebulized therapy

☐ Other: ______________

☐ None

9. Does the Child Have Any of the Following Pre-existing Conditions?

[Multiple-choice]

☐ Allergic rhinitis

☐ Asthma

☐ Heart disease

☐ Epilepsy

☐ Metabolic disorders

☐ Chronic gastrointestinal diseases

☐ Congenital conditions

☐ Tumors

☐ Rheumatic diseases

☐ Other: ______________

☐ None

☐ Febrile convulsions

10. Has the Child Completed Any COVID-19 Testing?

[Multiple-choice]

☐ Antigen positive

☐ Nucleic acid positive

☐ Not tested

☐ Antigen or nucleic acid positive in cohabitants within one week before or after onset

☐ Other: ______________

11. Days Until Symptoms Completely Disappeared:

[Open-ended]

---

12. Symptoms After One Week of Illness:

[Multiple-choice]

☐ Cough

☐ Hoarseness

☐ Chest tightness

☐ Chest pain

☐ Headache

☐ Fatigue

☐ Loss of appetite

☐ Diarrhea

☐ Abdominal pain

☐ Other: ______________

☐ None

13. Are You Willing to Accept Follow-up for Potential Long-Term Effects of COVID-19 in Children?

[Single-choice]

○ Yes (Please leave your phone number) ______________

○ No

14. Location of the Child's Residence:

[Single-choice]

○ Beijing

○ Shanghai

○ Guangzhou

○ Other Province: ______________

15. Date of the Child's First Symptoms of COVID-19:

[Open-ended]

---

16. During the First COVID-19 Infection, Were There Any Symptomatic Co-habitants?

[Multiple-choice]

☐ No

☐ Yes

17. Did the Child Experience a Second COVID-19 Infection?

[Single-choice]

○ No (Please skip to the end of the questionnaire and submit)

○ Yes, after complete recovery, the child had new symptoms such as fever or sore throat, but no new COVID-19 test was conducted

○ Yes

18. Date of the Child's Second COVID-19 Infection Symptoms:

[Open-ended]
